# A case–control study of agricultural and behavioral factors associated with leptospirosis in Vietnam

**DOI:** 10.1186/s12879-022-07561-6

**Published:** 2022-06-29

**Authors:** Luu Phuong Dung, Pham Thanh Hai, Luong Minh Hoa, Tran Ngoc Phuong Mai, Nguyen Thi My Hanh, Phan Dang Than, Van Dinh Tran, Nguyen Tu Quyet, Hoang Hai, Do Bich Ngoc, Nguyen Thị Thu, Le Thi Phuong Mai

**Affiliations:** 1 Department of Public Health, National Institute of Hygiene and Epidemiology, 1 Yersin Street, Hai Ba Trung District, Hanoi, Vietnam; 2 Hanoi Population and Family Planning Branch, Hanoi, Vietnam

**Keywords:** Human leptospirosis, Agricultural factors, Behavior factors, Risk factors, Vietnam

## Abstract

**Introduction:**

Leptospirosis is a neglected disease in Vietnam. Until now, there has been limited knowledge about risk factors of this disease in Vietnam. The study was carried out to identify agricultural and behavioral factors associated with the transmission of leptospirosis in Vietnam.

**Methods:**

This matched retrospective hospital-community-based case–control study was conducted from 1 October 2018 to 31 October 2019. We recruited cases from 11 selected government hospitals in three provinces of Vietnam, while controls were selected from the same communes of cases and matched by age (± 2 years) and sex. Microscopic agglutination test (MAT) and enzyme-linked immunosorbent assay (ELISA) were applied to determine confirmed cases, while only MAT was used to identify controls with a single high MAT titer < 1:100.

**Results:**

504 participants (252 cases and 252 controls) were identified. Cultivating (OR 2.83, CI 1.38–5.79), animal farming (OR 8.26, CI 2.24–30.52), pig owners (OR 10.48, CI 5.05–21.73), cat owners (OR 2.62, CI 1.49–4.61) and drinking unboiled water (OR 1.72, CI 1.14 –2.59, p = 0.010) were significantly associated with human leptospirosis in Vietnam. Hand washing after farming/ gardening (OR 0.57, CI 0.38–0.86, p = 0.007) and bathing after farming, gardening, contact with cattle and poultry (OR 0.33, CI 0.19–0.58, p = 0.000) were determined as protective factors for this disease.

**Conclusions:**

In short, the case–control study has revealed the risks in agricultural and animal practices and protective behavioral factors related to human leptospirosis in Vietnam. The findings suggested promotion of communication and health education programs targeting health behaviors in daily life and agricultural practices. Using personal protective equipment such as gowns, gloves, and boots during agricultural practices, especially cultivating and animal farming, is most recommended.

**Supplementary Information:**

The online version contains supplementary material available at 10.1186/s12879-022-07561-6.

## Introduction

Leptospirosis is a zoonotic bacterial disease recognized as a public health problem around the world. Most outbreaks occur in tropical and subtropical areas [1, 2]. Annually, leptospirosis was responsible for 1.03 million cases (95% CI 434,000–1,750,000) and 58,900 deaths (95% CI 23,800–95,900) worldwide [3]. Vietnam is considered an endemic area of leptospirosis as the incidence of disease has been recorded in many geographic areas nationwide [4]. Recent studies in several areas in Vietnam during the 1990 and 2000s found a significant presence of leptospirosis with seroprevalence ranging from more than 10–80%, depending on location and study population [5, 6]. However, a retrospective study based on 369 cases officially reported from 2002 to 2011 across the country revealed that the average annual incidence of leptospirosis was 0.05 per 100,000 inhabitants, which was much lower than that of neighboring countries [7–9]. Similarly, none or less than 20 cases were annually reported in the Infectious Disease Statistic Yearbook from 2014 to 2018 indicating leptospirosis underreported in Vietnam [10–14].

A number of risk factors for getting leptospirosis were identified in many publications elsewhere, in which factors related to behavioral and agricultural characteristics such as being a farmer, using open water source, health practices are significant in developing countries [15–20].

Most studies on *Leptospira* in Vietnam focused only on the seroprevalence of the pathogen, but not risk factors for disease infection [5, 6, 21]. Several cross-sectional studies addressed the relationship between leptospirosis and some major occupational groups such as farmers, slaughterhouses, animal raisers, and personal behaviors, such as not wearing personal protective equipment during farm work, swimming and wading in rivers, and walking barefoot. However, none of these were case-controlled studies, raising caution regarding their proposed recommendations [22]. Vietnam is still an agricultural country with 65% of inhabitants living in rural areas and 40% of the national workforce employed in agriculture, which increases the risk of *Leptospira* infection [23]. Nevertheless, many publications have addressed risk factors, to our knowledge, it is the first case–control study focusing on risk factors for leptospirosis in Vietnam. Our study aims to identify the main agricultural factors associated with acute leptospirosis transmission in Vietnam, which, in turn, may help to drive public health policy to improve preventions of disease.

## Methods

### Study setting

The case–control study was undertaken through the recruitment of clinically suspected patients from 11 public hospitals in Thai Binh, Ha Tinh and Can Tho provinces, which are located in the North, Center and South of Vietnam, respectively. The provinces selected in the study are in the populous group, generally, and among high population density groups in each region, particularly [24, 25]. These provinces also are among those that experience flooding or intensive agriculture, and most households live on agriculture [24–26]. These factors are likely to be an increased association with leptospirosis.

### Study design

**Fig. 1:**
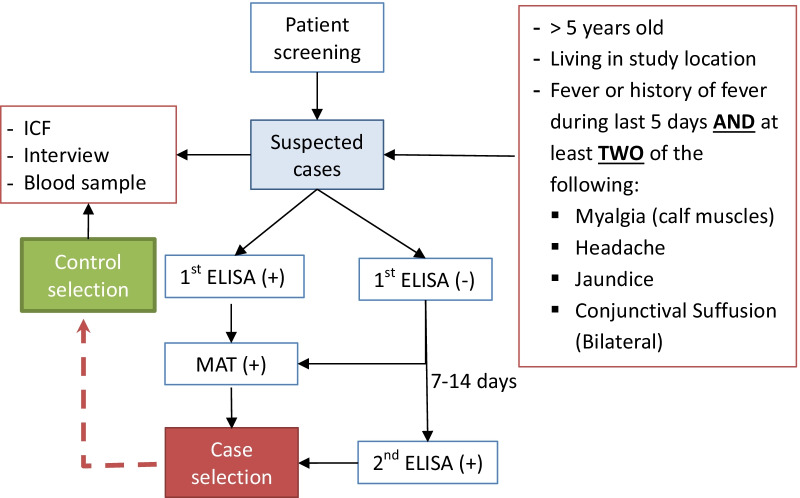
Process of case and control selection and data collection

The study design was a matched, retrospective, hospital-community-based case–control conducted from 1st October 2018 to 31st October 2019. All clinically suspected patients admitted to outpatient and inpatient departments of 11 selected government hospitals during the study period were screened as illustrated in Fig. 1 based on the selection criteria described below.

### Selection criteria of the cases with reference to WHO guidance [27]

**Table:** 

1.Children > 5 years old and adult patients admitted to outpatient and inpatient departments in the eleven selected public hospitals from October 2018 to October 2019, AND
2.Living in study areas for at least one month before recruitment, AND
3.Presenting with clinical signs suggesting leptospirosis (fever or history of fever within the last 5 days and had at least 2 of the following symptoms: myalgia, headache, jaundice, and conjunctival suffusion), AND
4.Either a fourfold rise in titer between the two consecutive anti-leptospiral IgM ELISA reactions in acute and convalescent-phase samples with titer ≥ 20 IU/ml; OR with a single high MAT titer ≥ 100*, AND
5.Provided written informed consent

*ELISA* enzyme-linked immunosorbent assay, *IgM* immunoglobulin M; *MAT* microscopic agglutination test

*Based on the epidemiological characteristics of leptospirosis in Vietnam and referring to previous studies [28–31], seropositivity was defined from the titer at a 1:100 dilution

### Selection criteria of the controls

Controls were recruited from the same commune of cases and matched by age (± 2 years) and sex. Controls also did not have symptom in the 10 days prior to enrollment and were negative with anti-leptospiral antibodies using the MAT laboratory technique.

### Blood sampling and data collection

A pretested structured questionnaire designed to collect exposure-related information from both cases and controls was administered by a doctor specially trained by the service's senior staff. Questionnaires were completed on the same day as hospital admission (case studies) or on approach (controls).

#### Collection of cases’ information

After obtaining informed consent, a trained member of the study team collected standardized clinical history and risk factor information from the recruited patients. Risk factor information included questions on sociodemographic characteristics, household living conditions, economic status, and risk behaviors. If the study participant was a child under 18 years of age, questionnaire was based on interviews with parent(s) or caregiver(s).

Each participant was asked for two samples of 3 ml-venous blood each. The first sample was collected at the time of recruitment. The sample, then, was centrifuged at 500–1000 ×*g* for 10 min. The resulting serum was stored at − 200 °C then transported to the National Institute of Hygiene and Epidemiology (NIHE), Hanoi, Vietnam for laboratory testing. Participants were asked to return 2 weeks post-enrollment for re-examination and the second sampling. Local health workers within the research team were responsible for reminding patients to return for follow-up care. After 3 reminder calls, if the patient did not return, the patient was excluded from the study.

#### Collecting control’s information

After obtaining informed consent, information was collected from control subjects using the same questionnaires as described for case subjects. As mentioned above, if the selected controls were children (less than 18 years), the guardians were assigned to interview.

Three milliliters venous blood was collected from control subject at the time of recruitment then stored and transported as described for case subjects. Negative MAT with 1:100 dilution was applied for the selection of the control.

### Data analysis

Data were entered in duplicate using Epi-Data 3.1 software to exclude possible typing errors. Statistical analyses were carried out with IBM SPSS for Windows, version 23.0. In the first step, bivariate logistic regression was performed to examine associations between acute leptospirosis and risk factors. After that, a stepwise backwards elimination was carried out by including all variables as the consideration of interactions between factors in bivariate logistic regression. The highest p-value variables, one after the other, were removed until all remaining variables in the model have a p-value smaller than 0.05 or until no variable was left in the model. Odds ratio (OR) and 95% confidence intervals (CI) were calculated, the p-values were two sided and all p-values less than 0.05 were indicated statistical significance in all analyses.

### Research ethics

The research protocol was approved by the Institutional Ethics Committee of the National Institute of Hygiene and Epidemiology, Hanoi, Vietnam. Written informed consent was obtained from all study participants. If a participant is less than 18 years old, informed consent was obtained from the parent(s)/guardian(s), instead.

## Results

A total of 504 participants, 252 cases and 252 controls, were included the final data set. The mean age of all participants was 43.81 ± 19.08 years, ranging from 5 to 87 years. Males accounted for 40.9% (*n* = 206) of all participants.

### Occupations and agricultural practices

Result of our bivariate analysis indicated association between occupation and acute leptospirosis, except for traders and students/ pupils/young children. Farmers were at higher risk (OR 1.90, CI 1.33–2.71, p = 0.004), while non-agricultural workers, mainly garment and textile or construction categories, and officials were at lower risk (OR 0.33, CI 0.19–0.75, p = 0.001 and OR 0.37, CI 0.15–0.91, p = 0.029, respectively). No risk association was observed for traders and students/pupils/young children. All agricultural practices had a statistically significant association with leptospirosis, with the highest risk at animal farming (OR 16.13, CI 4.92–52.83, p = 0.000), and the lowest risk at work on the rice field (OR 1.49, CI 1.53–3.88, p = 0.032). Additionally, being animal owners, except dog owners, showed a significant association with leptospirosis. However, no significant association was observed between rodents inside or outside the house and leptospirosis (Table 1).

**Table 1:** Bivariate logistic regression analysis of occupations and agricultural practices

Variable	Cases (n = 252)	Controls (n = 252)	OR (95% CI)	p
**Occupation**				
Farmers	153 (60.7%)	113 (44.8%)	1.90 (1.33–2.71)	0.004
Workers	20 (7.9%)	52 (20.6%)	0.33 (0.19–0.57)	0.0001
Officials	7 (2.8%)	18 (7.1%)	0.37 (0.15–0.91)	0.029
Traders	10 (4.0%)	7 (2.8%)	1.44 (0.54–3.86)	0.461
Students/Pupils/Young children	29 (11.5%)	26 (10.3%)	1.13 (0.64–1.98)	0.668
**Agricultural practices**				
Working in the rice field	126 (50.0%)	101 (40.1%)	1.49 (1.05–2.13)	0.032
Cultivating	56 (22.2%)	14 (5.6%)	4.86 (2.62–8.99)	0.000
Animal farming	41 (16.3%)	3 (1.2%)	16.13 (4.92–52.83)	0.000
**Being animal owners**				
Cattle owners	66 (26.2%)	32 (12.7%)	2.44 (1.53–3.88)	0.000
Pig owners	95 (37.7%)	13 (5.2%)	11.12 (6.03–20.54)	0.000
Dog owners	157 (62.3%)	139 (55.2%)	1.34 (0.94–1.92)	0.124
Cat owners	94 (37.3%)	29 (11.6%)	4.55 (2.86–7.24)	0.000
**Rodent exposure**				
Rodents seen inside the house	71 (28.2%)	58 (23.0%)	1.31 (0.88–1.96)	0.221
Rodents seen outside the house	32 (12.7%)	22 (8.7%)	1.52 (0.86–2.71)	0.153

In the multiple regression analysis, only cultivating (OR 2.83, CI 1.38–5.79), animal farming (OR 8.26, CI 2.24–30.52), pig owners (OR 10.48, CI 5.05–21.73) and cat owners (OR 2.62, CI 1.49–4.61) were independent variables remained, suggesting that these practices represent main risks for increased odds of leptospirosis (Table 2).

**Table 2:** Multiple logistic regression analysis of occupations and agricultural practices

Variable	OR (95% CI)	p
Cultivating	2.83 (1.38–5.79)	0.004
Animal farming	8.26 (2.24–30.52)	0.002
Pig owners	10.48 (5.05–21.73)	0.000
Cat owners	2.62 (1.49–4.61)	0.001

### Behavioral factors

Table 3 demonstrated the associations of behavioral factors with leptospirosis status, including risks and protective factors. Hand washing after using the toilet and agriculture or gardening indicated 0.63 (OR = 0.37, CI 0.22–0.62, p = 0.000) and 0.62 (OR = 0.38, CI 0.27–0.55, p = 0.000), respectively, less likely to get the disease. Similarly, bathing after farming, gardening, cattle/poultry contact and using gloves during livestock or farming activities have also been shown to be protective factors, with 0.74 (OR = 0.26, CI 0.16–0.44, p = 0.000) and 0.45 (OR = 0.55, CI 0.37–0.81, p = 0.002) lower odds of acquiring leptospirosis. On the contrary, drinking unboiled water significantly increased the risk of leptospirosis (OR = 2.08, CI 1.42–3.05, p < 0.05). The remaining practices shown no significant association with the disease.

**Table 3:** Bivariate logistic regression analysis of behavioral factors

Variable	Cases (n = 252)	Controls (n = 252)	OR (95% CI)	p
Hand washing after using toilet	198 (78.6%)	229 (90.9%)	0.37 (0.22–0.62)	0.000
Hand washing after farming/gardening	85 (33.7%)	144 (57.1%)	0.38 (0.27–0.55)	0.000
Hand washing before eating	165 (65.5%)	158 (62.7%)	1.13 (0.78–1.62)	0.516
Hand washing after bathing the livestock or assisting them to breed	93 (36.9%)	1115 (45.6%)	0.69 (0.49–1.00)	0.050
Hand washing after contacting domestic animals	102 (40.5%)	122 (48.4%)	0.73 (0.51–1.03)	0.073
Bathing after farming, gardening, cattle/poultry contact	185 (73.4%)	230 (91.3%)	0.26 (0.16–0.44)	0.000
Using gloves/boots for farming, gardening, livestock/poultry contact	158 (62.7%)	190 (75.4%)	0.55 (0.37–0.81)	0.002
Walking barefoot	171 (67.9%)	171 (67.9%)	–	1
Participating in physical activities	198 (78.6%)	183 (72.6%)	1.38 (0.92–2.01)	0.120
Participating in water sports	21 (8.3%)	30 (11.9%)	0.67 (0.37–1.21)	0.184
Drinking unboiled water	102 (40.5%)	62 (24.6%)	2.08 (1.42–3.05)	0.000
Eating uncooked food	57 (22.6%)	54 (21.4%)	1.07 (0.70–1.63)	0.747

The multiple logistic regression analysis of behavior risk factors indicated three protective factors—hand washing after using toilet (OR 0.39, CI 0.23–0.68, p = 0.001), hand washing after farming/gardening (OR 0.57, CI 0.38–0.86, p = 0.007) and bathing after farming, gardening, contacting with cattle and poultry (OR 0.33, CI 0.19–0.58, p = 0.000). Each of these behaviors was significantly associated with reduction in odds of leptospirosis. In contrast, drinking unboiled water (OR 1.72, CI 1.14 –2.59, p = 0.010) increased the risk of having leptospirosis (Table 4).

**Table 4:** Multiple logistic regression analysis of behavioral factors

Variable	OR (95% CI)	p
Hand washing after using toilet	0.39 (0.23–0.68)	0.001
Hand washing after farming / gardening	0.57 (0.38–0.86)	0.007
Bathing after farming, gardening, cattle / poultry contact	0.33 (0.19–0.58)	0.000
Drinking unboiled water	1.72 (1.14–2.59)	0.010

## Discussion

Agricultural activities in Vietnam account for over 70% of the national workforce. Activities such as rice and fruit farming are typically associated with environmental conditions that are suitable for the survival of *Leptospira* spp. If no appropriate preventive measures, Vietnamese farmers who engage in livestock and cultivating would be at increased risk of getting leptospirosis [32, 33]. These occupations should deserve more attention in Vietnam. Our findings indicated that farmers and agricultural activities, especially cultivation, were positively associated with a higher risk of leptospirosis compared to other occupational groups. These findings are consistent with previous perceptions of occupational groups at risk for leptospirosis. For example, studies conducted in Thailand, Indonesia, and the Asia–Pacific region reported that agricultural workers are the main occupational risk groups for leptospirosis [34, 35]. Farmers can become infected after contact with the urine of infected animals or with leptospires in the wet environment during their daily activities [36, 37]. As a result, the prevalence of leptospirosis among farmers often is higher than in other groups. As shown by a study from Sri Lanka, the weekly report of occupational exposure among the farmers indicated 43.5% of leptospirosis patients had been engaged in paddy fields [38]. In Iran, 36.1% of leptospirosis patients belong to a farmer group, which had higher seroprevalence in comparison to other groups [39].

In addition, our study also identified other risks related of leptospirosis to swine or cat raising. The previous studies indicated pigs and cats as reservoirs of *Leptospira* throughout the world and Vietnam [40–44]. Various serovars, such as Castellonis and Patoc, Tarassovi Mitis, Australis, Javanica, and Autumnalis in swine; and Javanica, Louisiana, Hebdomadis, and Castellonis in cats, were also found in *Leptospira* patients [21, 45–47]. Our study found no association between the risk of leptospirosis and exposure with cattle, dogs, and rats, which was slightly different from previous knowledge, such as studies in Canada, Brazil found frequent and close rat exposure, particularly Norway rats, increased risk of infection, while serovars found in rodents and strain typing confirmed rodents as reservoirs for human leptospirosis studies in Italy, Southeast Asia [35, 48–51].

According to the World Health Organization (WHO), some behaviors could help prevent exposure to *Leptospira*, while other behaviors could put people at higher risk [52]. Exposure to *Leptospira* contaminated environment is a risk of disease transmission [53], as a result, washing hands, bathing before eating/ after contacting the source of infection, or eating cooked and drinking boiling water were recommended by the WHO as preventative measures [52]. A 1998 study in Missouri indicated that washing hands after participating in livestock activities was a protective factor for leptospirosis (OR 0.2, CI, 0.03—0.81) [54]. Similarly, we found that good behavioral practices were significantly associated with a reduction of the leptospirosis risk. Specifically, protective factors were washing hands after using the toilet (OR 0.39, CI 0.23–0.68, p = 0.001), washing hands after farming, gardening (OR 0.57, CI 0.38–0.86, p = 0.007) or taking a bath after participating in livestock farming (0.33, CI 0.19–0.58, p = 0.000). Contrarily, drinking unboiled water was a factor related to increased odds of leptospirosis (OR 1.72, CI 1.14 –2.59, p = 0.010). Additionally, the use of protective gear is also considered a protective factor for this disease [52]. Several studies also generated the same findings. Brown et al. in 2011 indicated that the absence of personal protective gear during participation in agricultural activities is significantly associated with human leptospirosis because workers are exposed to contaminated feces, blood, and other secretions [55]. The similar findings were synthesized by Sakundarno et al*.* in a review of 34 studies conducted in the Indonesia and other countries in Asia–Pacific region in 2014 [53]. In our study, however, wearing personal protective equipment as a protective factor was only observed in the bivariate logistic regression, but not in the multivariate model (Additional file 1).

Our study had some limitations. First, we applied lower titer dilution of MAT test for confirmed cases as the WHO guidelines [27]. It is because an unknown febrile case with positive MAT at a dilution of 1:100 was defined and treated as a confirmed case of leptospirosis in health facilities in Vietnam. On the other hand, prior studies used a similar titer level for identifying risk factors. For instance, a study in Uganda in 359 non-pregnant adults used MAT titer of > 1:100 against any serovars to define seropositive case for risk factor analysis. This titer level was also observed in a study conducted by the Department of Maladies Infectieuses et Tropicales, Hôpital de la Pitié‐Salpêtrière, Paris in 15 travel‐related leptospirosis cases [30, 31]. Second, our study was conducted only in three provinces, and may not representative of risk factors nationwide, which may have differed in the epidemiology of *Leptospira*.

In conclusion, the case–control study has revealed the risks in agricultural and animal practices and protective behavioral factors related to human leptospirosis in Vietnam. The findings suggested promotion of communication and health education programs targeting health behaviors in daily life and agricultural practices. Using personal protective equipment such as gowns, gloves, and boots during agricultural practices, especially cultivating and animal farming is highly recommended.

## Supplementary Information

**Additional file 1.** Study dataset.

## Data Availability

All data generated or analyzed during this study are included in this published article with the file name “210701—R1—FINAL—CASE CONTROL”.
